# Linearly polarized photoluminescence of InGaN quantum disks embedded in GaN nanorods

**DOI:** 10.1038/s41598-018-26642-8

**Published:** 2018-05-25

**Authors:** Youngsin Park, Christopher C. S. Chan, Luke Nuttall, Tim J. Puchtler, Robert A. Taylor, Nammee Kim, Yongcheol Jo, Hyunsik Im

**Affiliations:** 1 School of Natural Science, Ulsan National Institute of Science and Technology (UNIST), Ulsan, 44919 Korea; 2 Clarendon Laboratory, Department of Physics, University of Oxford, Oxford, OX1 3PU UK; 3 Department of Physics, Soongsil University, Seoul, 06978 Korea; 4 Division of Physics and Semiconductor Science, Dongguk University, Seoul, 04620 Korea; 5 Department of Physics, Hong Kong University of Science and Technology, Clear Water Bay, Hong Kong China

## Abstract

We have investigated the emission from InGaN/GaN quantum disks grown on the tip of GaN nanorods. The emission at 3.21 eV from the InGaN quantum disk doesn’t show a Stark shift, and it is linearly polarized when excited perpendicular to the growth direction. The degree of linear polarization is about 39.3% due to the anisotropy of the nanostructures. In order to characterize a single nanostructure, the quantum disks were dispersed on a SiO_2_ substrate patterned with a metal reference grid. By rotating the excitation polarization angle from parallel to perpendicular relative to the nanorods, the variation of overall PL for the 3.21 eV peak was recorded and it clearly showed the degree of linear polarization (DLP) of 51.5%.

## Introduction

Wurtzite GaN and its family of compounds such as InN, InGaN etc. have a strong piezoelectric field along the crystal *c*-axis^1^, which causes a quantum confined Stark effect (QCSE). This can induce harmful effects to device performance due to the shift of the emission energy and the separation of the electron-hole wavefunction in space. However, hetero nanostructures like InGaN/GaN quantum disks (QDisk) can reduce the QCSE by lateral quantum confinement and strain relaxation. Optical polarization studies of one-dimensional nanostructures such as nanowires and nanorods have been widely investigated^2–4^. Photon antibunching and linearly polarized emission were demonstrated from InGaN/GaN quantum dots (QDs) and attributed to the valence-band mixing by the lateral anisotropy of the wurtzite group-III nitrides^5–7^. Though there is no complete solution, so far, several efforts on the position and polarization control of QDs have been reported^8,9^. Additional polarization was reported at the nonpolar heterostructures caused by the in-plane anisotropy of the band structure, resulting in polarization of photo- and electro-luminescence^10–12^.

Here we report the optical polarization of InGaN/GaN QDisks with a diameter of 150 nm grown on GaN nanorods. The optical properties were characterized by micro photoluminescence (PL) and time-resolved micro-photoluminescence (TRPL). The PL peak around 3.21 eV originating from the InGaN QDisks doesn’t show the Stark shift and polarized emission with a degree of linear polarization (DLP) of 39.3% was observed when the nanorods were excited orthogonally to their growth axis due to crystal anisotropy. In order to characterize a single nanostructure, the QDisks were spread on a reference marked SiO_2_ substrate.

## Results and Discussion

**Figure 1:**
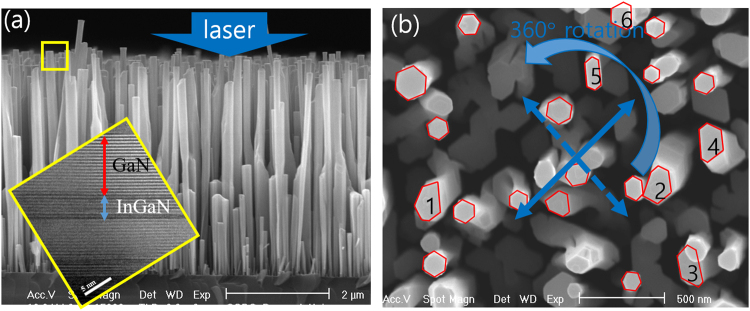
Scanning electron microscopy image of the InGaN/GaN quantum disks grown on GaN nanorods in (**a**) cross-sectional view and (**b**) plan-view. Inset in (**a**) depicts the transmission electron microscopy image of the InGaN/GaN disk region. The blue arrows in (**b**) represent the excitation laser direction by rotating 360°. The red hexagons are guides for the eye. The numbered nanostructures show nanorods of asymmetric shape.

Figure 1(a) shows the cross-sectional scanning electron microscopy (SEM) image of the InGaN/GaN quantum disk grown on GaN nanorods. The inset depicts the transmission electron microscopy (TEM) image of the InGaN/GaN region marked in the yellow squared region. The nanorods were aligned with a vertical direction on the Si substrate. Figure 1(b) show the plan view of the SEM image of the nanorods. The majority of nanorods exhibited a hexagonal shape, although some (numbered in the figure) show an elongated shape along a specific direction. The excitation laser direction for PL is an arbitrary.

**Figure 2:**
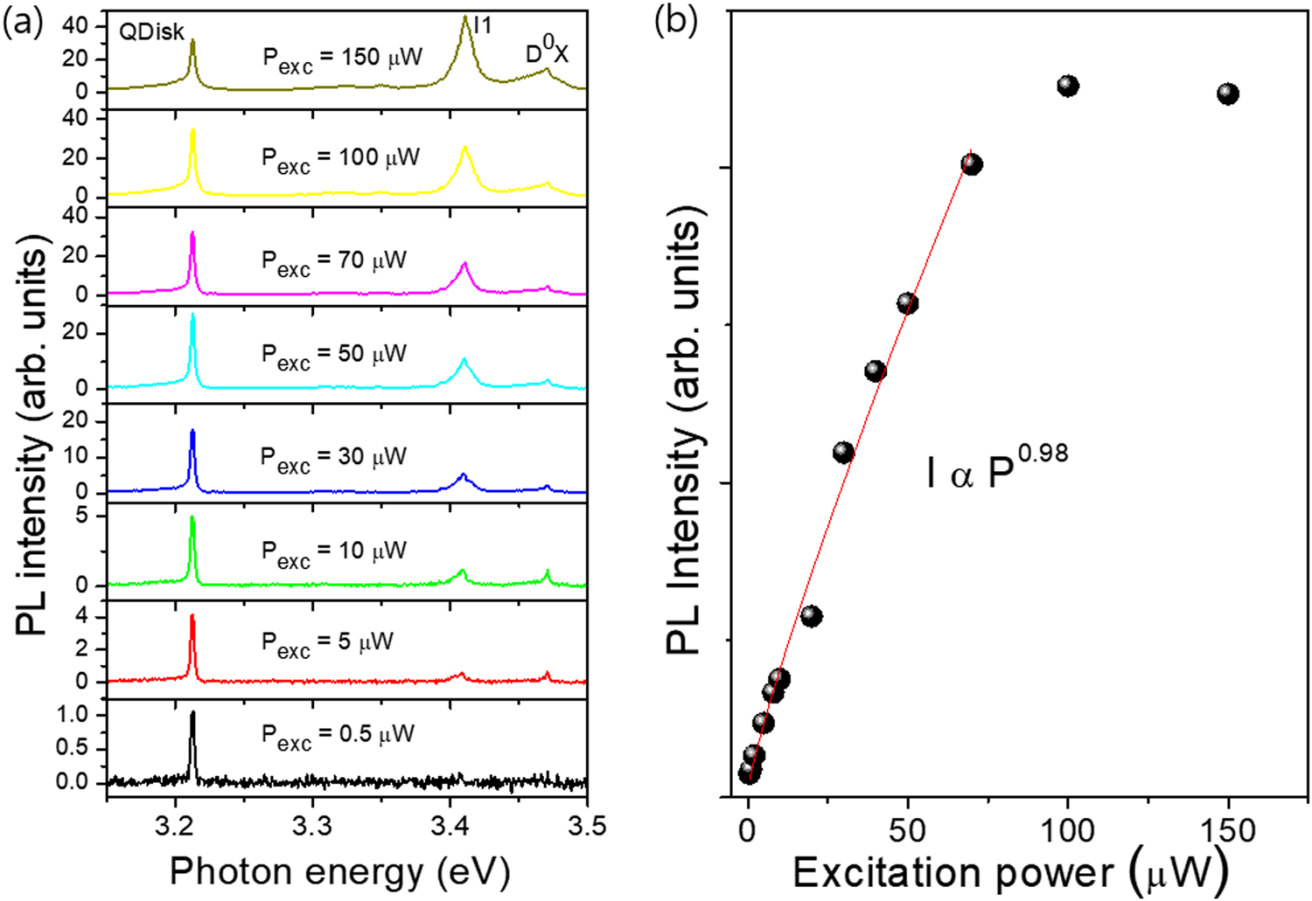
(**a**) Excitation power dependent PL of the InGaN/GaN on GaN nanorods. (**b**) The integrated PL intensity as a function of excitation power.

Figure 2(a) shows the excitation power-dependent PL spectra of the InGaN/GaN QDisks measured from above as shown in Fig. 1(b). Emission peaks attributed to donor bound exciton (D°X) near 3.47 eV, defect related emission (I_1_) near 3.41 eV, and quantum disk related emissions near 3.21 eV with a full width at half maximum of about 3 meV were observed. Our study was focused on the quantum disk related emission. Emission energy showed no dependence on excitation power, hence the sample exhibited no Stark shift, whereas the integrated PL intensity increases linearly with excitation power due to the excitonic emission as shown in Fig. 2(b). The PL shift due to QCSE is related to a decrease in localization energy from a lower degree of indium fluctuation and decrease in QCSE with lower indium content and a possibly a less strain. The PL intensity is fitted with the following relation I ∝ P^*α*^ where I is the PL intensity, P is the excitation laser power. The intensity increases linearly in the low excitation power region and saturates at around 80 μW. The PL peak intensity increases as follows, I α P^1.07^. Note that for excitonic emissions α = 1. Consecutive PL spectra of 5 s accumulation time were collected from the sample to establish the role of dislocations in spectral diffusion (Supporting information S1). The minimal temporal variation in the emission energy shows little spectral diffusion is present in this sample^13^.

**Figure 3:**
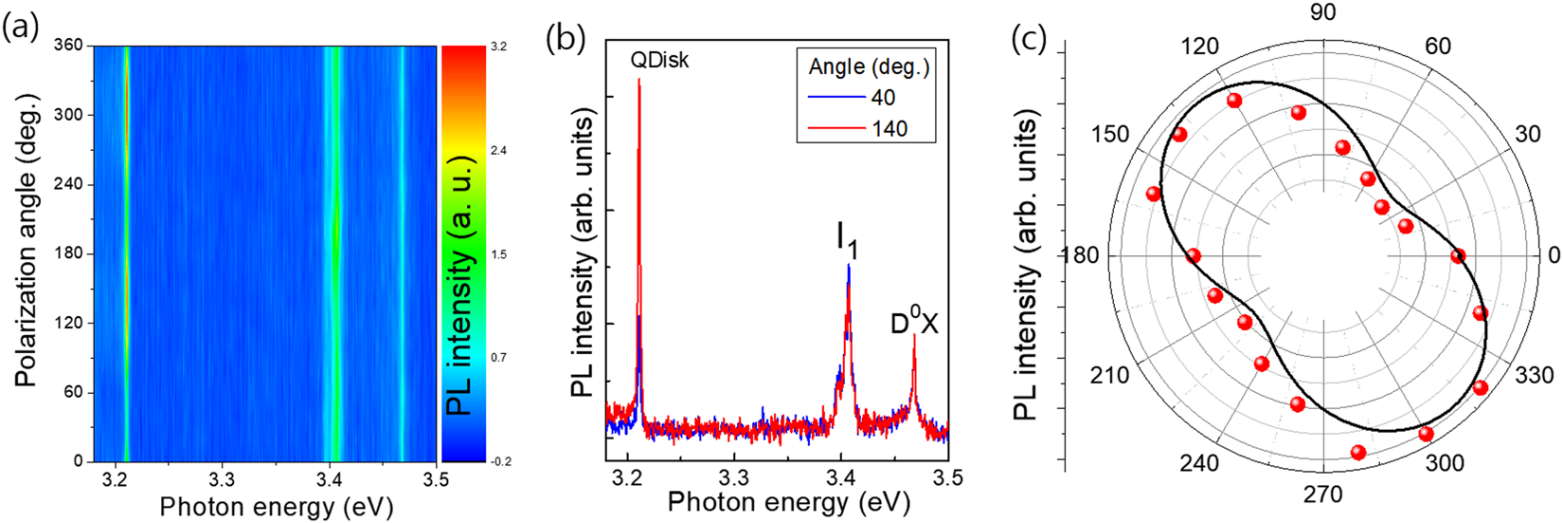
(**a**) PL spectra mapping of the InGaN/GaN nanostructures with different excitation angles. (**b**) The selected PL spectra taken at 40° and 140° with minimum and maximum PL intensities. (**c**) Integrated PL intensity for the 3.21 eV emission as a function of excitation angle.

Prior to measurement of the InGaN/GaN QDisk PL, we measured the polarized angle dependent PL for the GaN epilayer to symmetrically optimize the PL system (see Supporting information Fig. S2). Figure 3(a) shows the polarization dependent PL intensity plot of the InGaN QDisk grown on the GaN nanorods at an excitation power of 20 μW. The starting angle is arbitrary as shown in Fig. 1(b). Interestingly, whilst the PL intensities of the D°X near 3.47 eV and I_1_ near 3.41 eV are independent on the polarizer angle, that of the QDisk related emission near 3.21 eV shows a clear sinusoidal variation. Figure 3(b) shows the PL spectra at minimum (40°) and maximum (140°) intensities from the polarizer angle dependent PL, displaying linearly polarized emission.

Figure 3(c) shows the angular dependence of the integrated PL peak intensity (circles) as a function of the incident linear polarization angle. The polarization angles in Fig. 3(c) are relative to an arbitrary direction (where our linear analyzer started measuring). The data is fitted well by Malus’s law, *I*(θ) = *I*_min_ + *I*_max_ cos^2^ (*θ* − *θ*_0_), where θ is the of polarization angle and *I*_min_ and *I*_max_ are the minimum and maximum intensities, respectively. The degree of linear polarization (DLP), defined by (*I*_max_ − *I*_min_)/(*I*_max_ + *I*_min_), in the measured angular range is calculated to be ~39.3%. A large DLP up to 90% has been demonstrated in the GaN/AlN QDs owing to the frame of C3v symmetry of wurtzite III-nitrides, that is due to the anisotropy in the QD confining potential resulting in valence band mixing effects^5,14^. Note that the plan-view image of the nanorods (Fig. 1(b)) shows that many nanorods have a hexagonal shape, however, some have elongated hexagonal shapes with an arbitrary directions marked as a number in the image, which is in good agreement with the polarization shape. This is well consistent with that the polarization direction is parallel to the elongation^4,15^.

**Figure 4:**
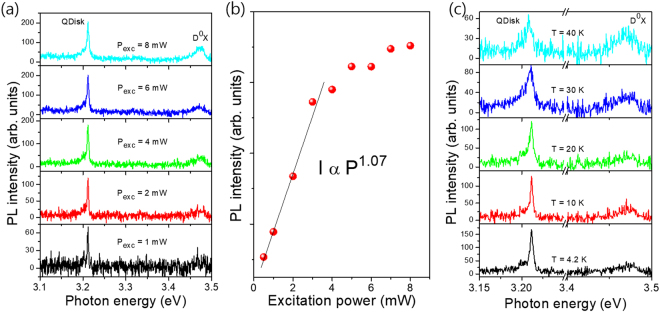
(**a**) Excitation power dependent PL of a single InGaN/GaN nanostructure prepared on a metal marked SiO_2_. (**b**) The integrated PL intensity as a function of excitation power. (**c**) Temperature dependent PL of the InGaN/GaN nanostructure.

In order to investigate the polarization of the individual nanorod structure, the QDisks grown on the GaN nanorods with a diameter of 150 nm were mechanically detached from the as grown substrate into an acetone solution by a brush and the solution was spread over the metal marked SiO_2_ wafer (Supporting information S3). Figure 4(a) shows the excitation power-dependent μ-PL spectra of the QDisks taken from a single nanorod. Differences compared to the non-dispersed nanorod μ-PL measurements can be seen clearly. The overall PL intensity is low due to the single nanorod excitation. As the excitation power is increased, the intensity of the QDisk emission line increases linearly as shown in Fig. 4(b). The confinement was confirmed by measuring the temperature dependent PL, which is showing that the PL energy is almost constant around low temperature as shown in Fig. 4(c). The full width at half maximum increases with increasing temperature.

**Figure 5:**
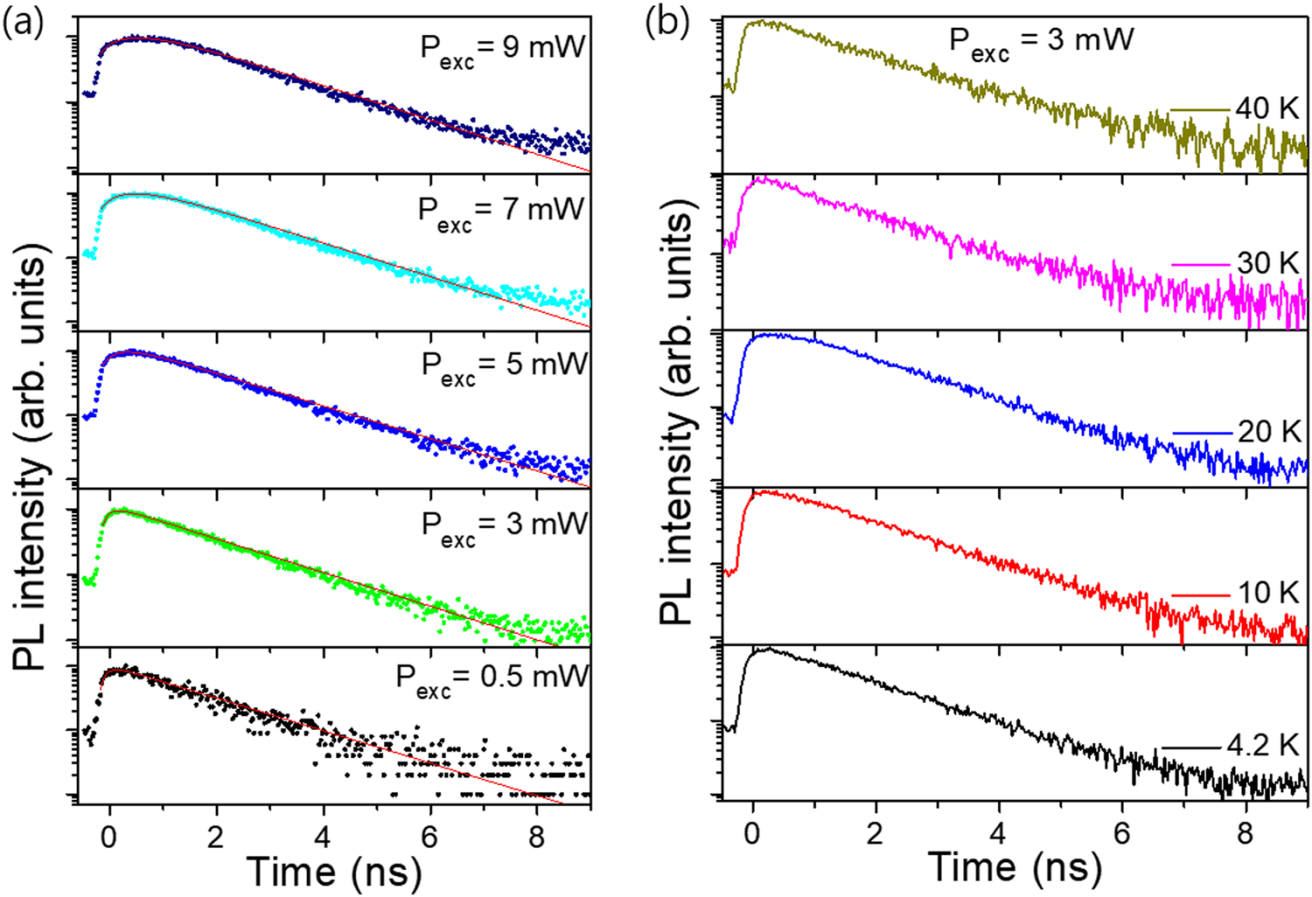
(**a**) Excitation power dependent time-resolved PL spectra measured at 4.2 K. All color dots and red solid lines are measured and fitted data, respectively. (**b**) Temperature dependent time-resolved PL spectra.

Figure 5(a) shows the excitation power dependent TRPL spectra of the 3.21 eV peak originating from the quantum disk emission measured at 4.2 K. The decay curve shows a single exponential component with a decay time of 1.45 ns at low excitation power which is comparable with a value of 2 ns in the InGaN/GaN QDisk^16^. This single exponential decay is mainly coming from the excitonic recombination^17,18^. In addition, the decay time doesn’t significantly change with increasing excitation power, indicating that there is no Stark effect influencing the exciton emission in the InGaN quantum disk. This supports the previous result of excitation power dependent PL which shows that the peak energy doesn’t change with excitation power (Fig. 4(a)). Note that at high powers the TRPL curve displays a rise-time immediately after excitation but before exponential decay because the carriers excited from GaN barrier recombine with D^0^X of the GaN and excess carriers move to InGaN QDisks. The system is modelled as having two populations of excitons: a high energy population (n_1_), and a low energy population (n_2_). Excitons in the first population decay non-radiatively into the second with a rate constant k_1_, then those excitons decay radiatively with a rate constant k_2_. This yields a system of two differential equations:

1 $${{\rm{dn}}}_{1}/{\rm{dt}}=-\,{{\rm{k}}}_{1}{{\rm{n}}}_{1}$$

2 $${{\rm{dn}}}_{2}/{\rm{dt}}={{\rm{k}}}_{1}{{\rm{n}}}_{1}-{{\rm{k}}}_{2}{{\rm{n}}}_{2}$$

These parameters (initial n_1_, initial n_2_, k_1_, and k_2_) are fitted to the TRPL data using the trust region reflective algorithm^19^ to perform least squares optimization. At each optimization step the system of differential equations is solved using a Runge-Kutta method of order 4(5)^20^ to give the function, n_2_(t), which is proportional to the PL intensity. This function is convolved with the instrument response function (IRF**)** and normalized before being compared to the real data.

Figure 5(b) shows the temperature dependent TRPL spectra from 4.2 K to 40 K. We used the excitation power of 3 mW to avoid thermal and carrier accumulation effects. Additional measurements were performed using 1 mW, in order to remove the thermal effect due to the laser heating, however, the decay time doesn’t change with temperature (Supporting information Fig. S4).

**Figure 6:**
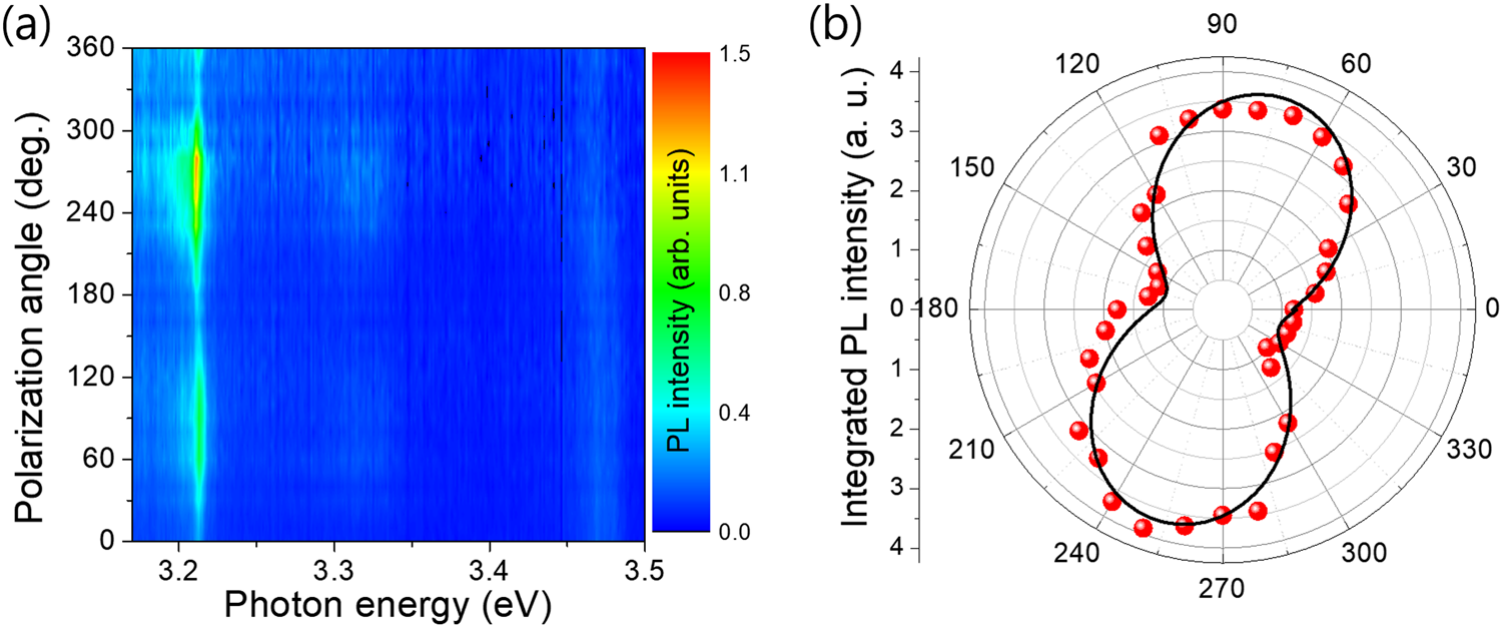
(**a**) Polarization angle dependent PL intensity plot of a single InGaN/GaN quantum disk dispersed on a pattern SiO_2_ substrate with different excitation angle. (**b**) Integrated PL intensity for the 3.21 eV emission as a function of excitation angle.

Figure 6(a) shows the PL polarization with different excitation angles. For the GaN nanorods, the D^0^X emission near 3.47 eV doesn’t show the polarization effect when we excited it along the parallel and perpendicular direction of the nanorods. Chen *et al*. reported that the maximum DLP occurs at the rod diameter of ~40 nm, and then decreases rapidly with increasing diameters due to the optical confinement effects in the size regime^3^. On the other hand, the polarized PL from the QDisk peak near 3.21 eV was observed by rotating the polarizer. The starting angle is arbitrary. Figure 6(b) shows the variation in PL intensity of the QDisk with polarization angle, which shows a DLP of about 51.5%. Though this value is lower compared to the previous reported values of InGaN QDs (~70%)^15,21^, the nanorods structures offer greater control of sample position. For comparison purposes, we carried out PL measurements for the InGaN/GaN nanostructure. A clear PL peak from quantum disks is clearly observed near 3.24 eV, as shown in Fig. S5 (Supporting information) and the emission shifts with increasing excitation power, indicating that the Stark shift is observed from the quantum disk emission. The PL intensity increases linearly and saturates with a slope of about 0.92 due to the excitonic emission of the InGaN/GaN quantum disks. As shown in Fig. S5(d) (Supporting information), the polarization is very weak with the DLP of less than 10%.

## Conclusions

In summary, we have investigated the emission from InGaN/GaN quantum disks grown on GaN nanorods. The emission at 3.21 eV from the InGaN quantum disk doesn’t show a power dependent Stark shift and shows linearly polarized behavior when we excite the nanostructure orthogonal to the growth direction. The degree of linear polarization is about 39.3% due to the anisotropy of the nanostructures. The degree of linear polarization increases to 51.5% when we excite a single InGaN quantum disk dispersed on a patterned SiO_2_ substrate.

## Methods

The GaN nanocolumns probed in this investigation were grown on Si (111) substrates under nitrogen rich conditions by rf-plasma assisted molecular beam epitaxy, the growth conditions of which can be found elsewhere^22–24^. The nanocolumns are typically ~150 nm in diameter and about ~4 μm in length. 5 InGaN QDisk layers with an indium content of 15% were grown on top of the columns, at reduced temperature (700 °C) to facilitate the incorporation of indium, before being capped with a thin (3 nm) layer of GaN.

A frequency-tripled femto-second Ti:sapphire laser (100 fs pulses at 76 MHz) operating at 266 nm was used to excite the nanorods in the micro-photoluminescence experiment. The sample was mounted in a continuous-flow helium cryostat, allowing the temperature to be controlled accurately at 4.2 K. A 36x reflecting objective lenz was mounted above the cryostat and the incident laser beam was focused to a spot size of ~2 μm^2^ and the PL signal was corrected. The luminescence was then directed to a spectrometer with a spectral resolution of ~700 μeV. The PL signal was recorded by using a cooled charge coupled device (CCD) detector. The laser spot size indicates that ~20 nanorods were excited at one time, and the size distribution of nanorods made it possible to find spectrally isolated emission from a single nanorod. Polarization properties were analyzed by rotating a linear polarizer over a range of 360°. The polarization rotator was located in front of the laser to vary the polarization of the incident laser. The measured PL polarization is corrected to compensate for the depolarization effects caused by the system, which mainly originate from the optics involved in the incident path and are determined by characterizing the beam polarization at the sample position.

## Electronic supplementary material

Supplementary information
