# Supplementary material for: Linearly polarized photoluminescence of InGaN quantum disks embedded in GaN nanorods
Source: Sci Rep. 2018 May 25;8:8124. doi: 10.1038/s41598-018-26642-8 (PMC5970171; doi:10.1038/s41598-018-26642-8)
Supplement: Supplementary file 1 — Supplementary information [file 41598_2018_26642_MOESM1_ESM.docx]

Supporting information on

Linearly polarized photoluminescence of InGaN quantum disks embedded in GaN nanorods

# Youngsin Park^1^, Christopher C. S. Chan^2,5^, Luke Nuttall^2^, Tim J. Puchtler^2^, Robert A. Taylor^2^, Nammee Kim^3^, Yongcheol Jo^4^, and Hyunsik Im^4^

^1^School of Natural Science, Ulsan National Institute of Science and Technology (UNIST), Ulsan 44919, Korea

^2^Clarendon Laboratory, Department of Physics, University of Oxford, Oxford, OX1 3PU, UK

^3^Department of Physics, Soongsil University, Seoul 06978, Korea

^4^Division of Physics and Semiconductor Science, Dongguk University, Seoul 04620, Korea

^5^Department of Physics, Hong Kong University of Science and Technology, Clear Water Bay, Hong Kong, China


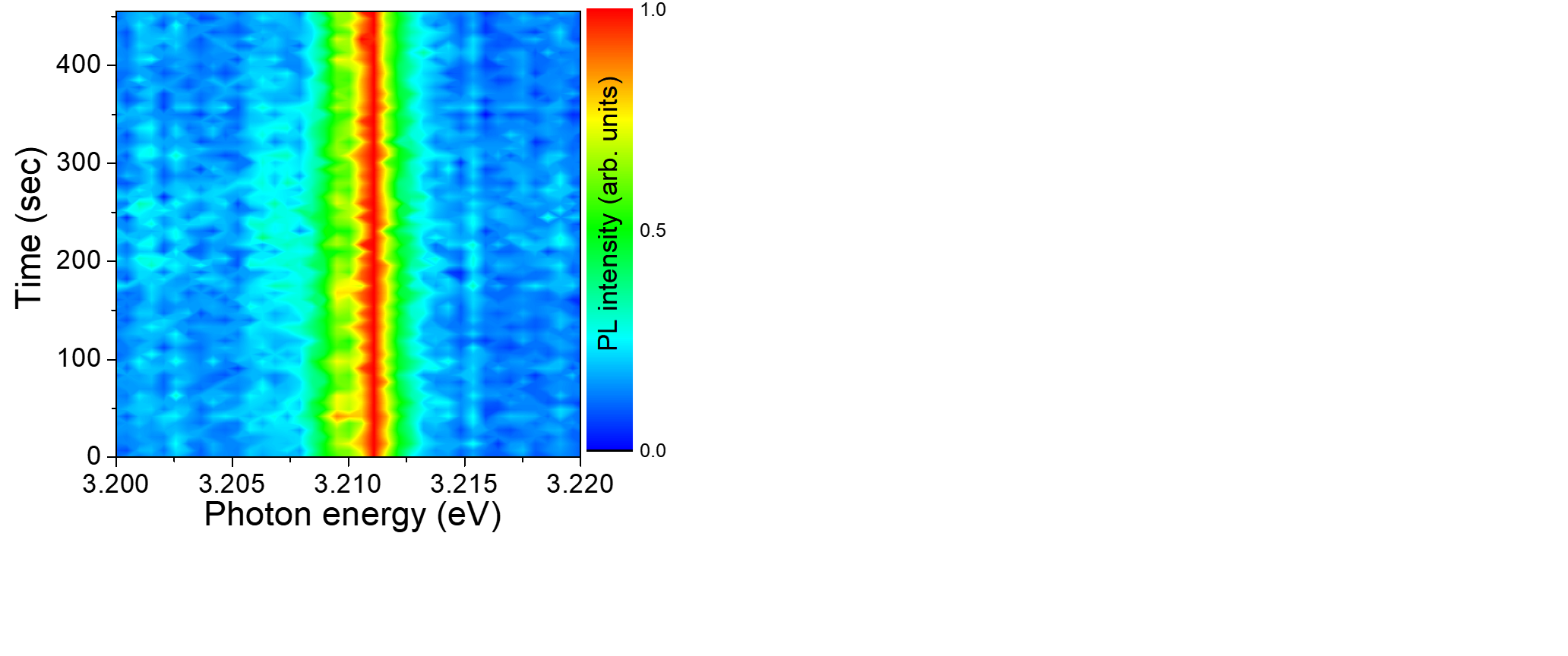


Figure S1. Temporal PL spectra of the InGaN/GaN emission.


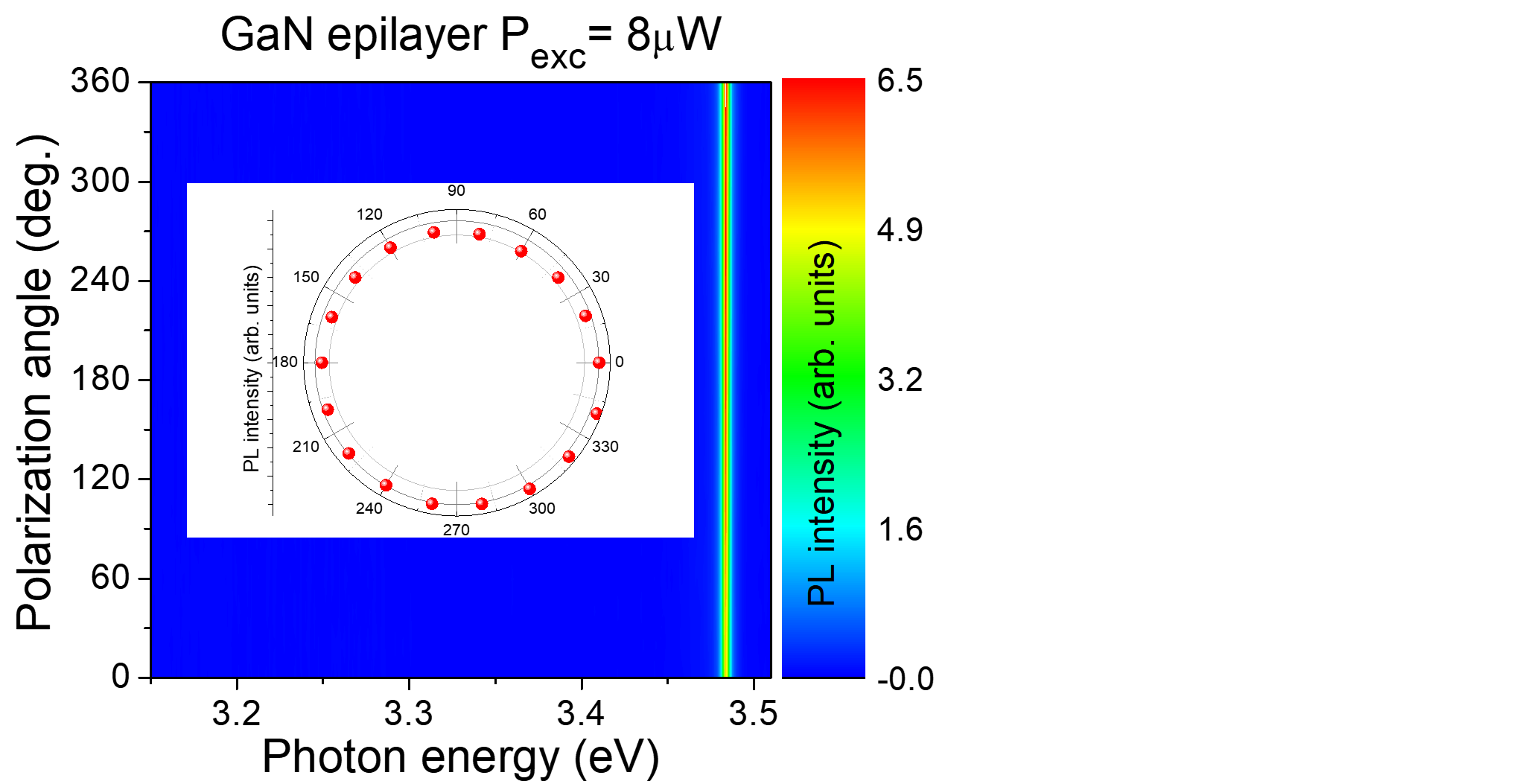


Figure S2. PL spectra of the GaN layer with different polarization angle of the laser. Inset depicts the integrated PL intensity as a function of excitation laser angle.


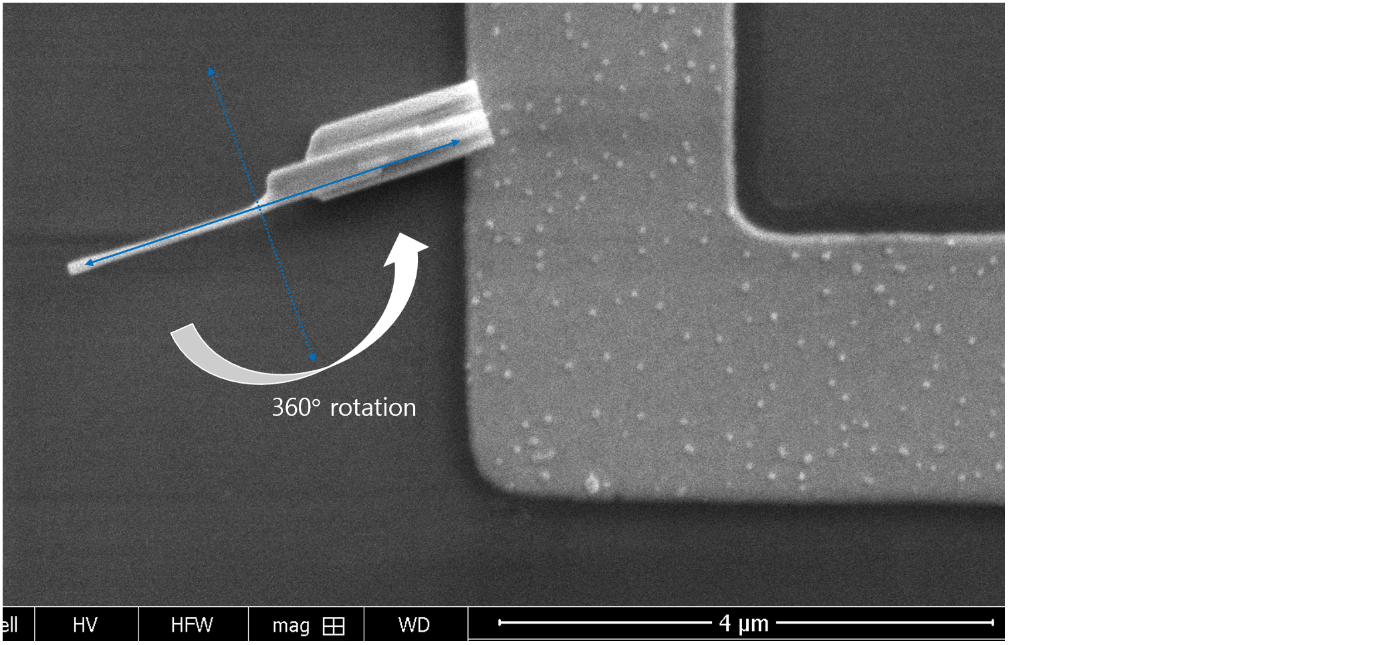


Figure S3. Scanning electron microscopy image of the InGaN/GaN nanostructure dispersed on a patterned SiO_2_ substrate. The blue arrows represent the excitation laser direction.


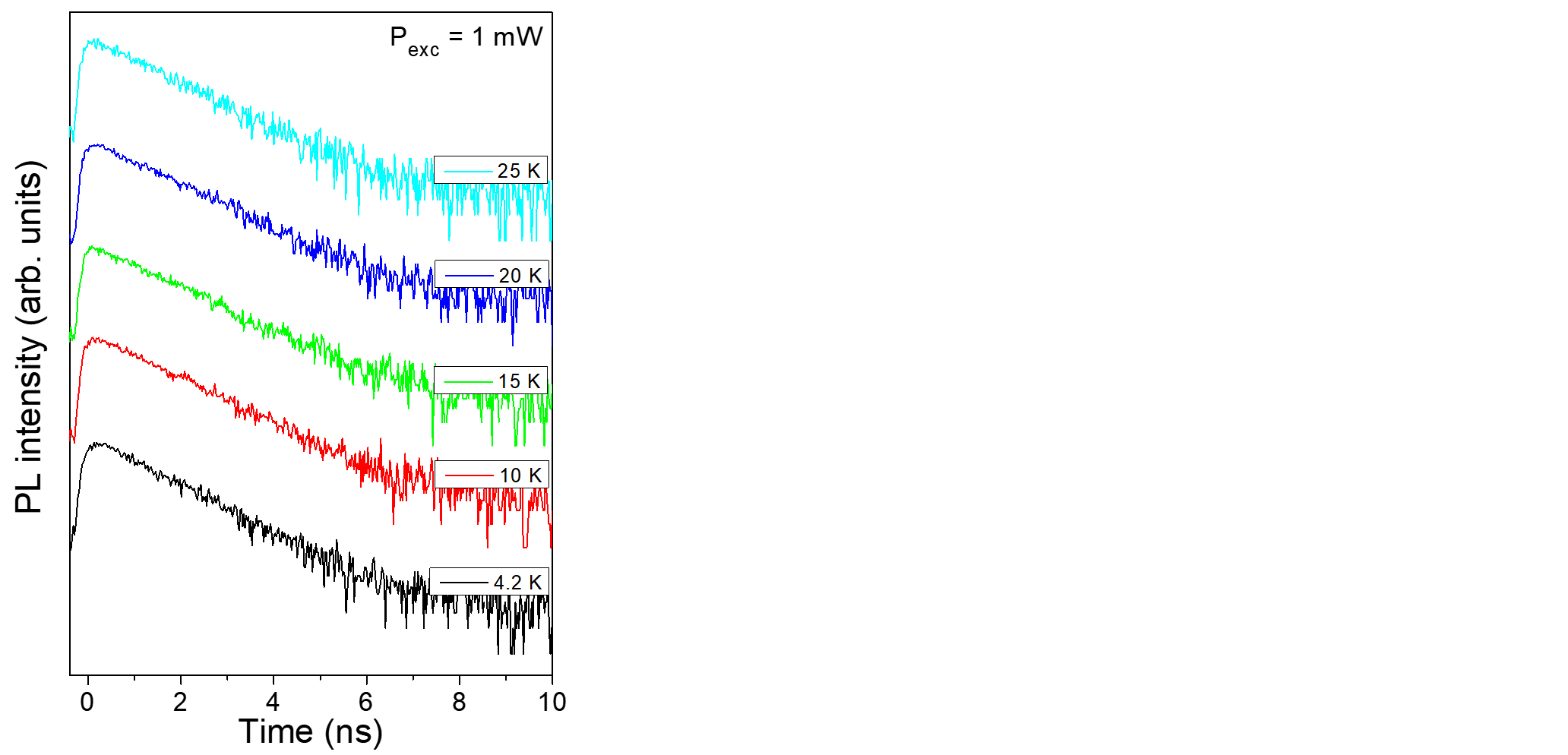


Figure S4. Temperature dependent time-resolved PL spectra measured at an excitation power of 1 mW. The decay time doesn’t change with temperature.


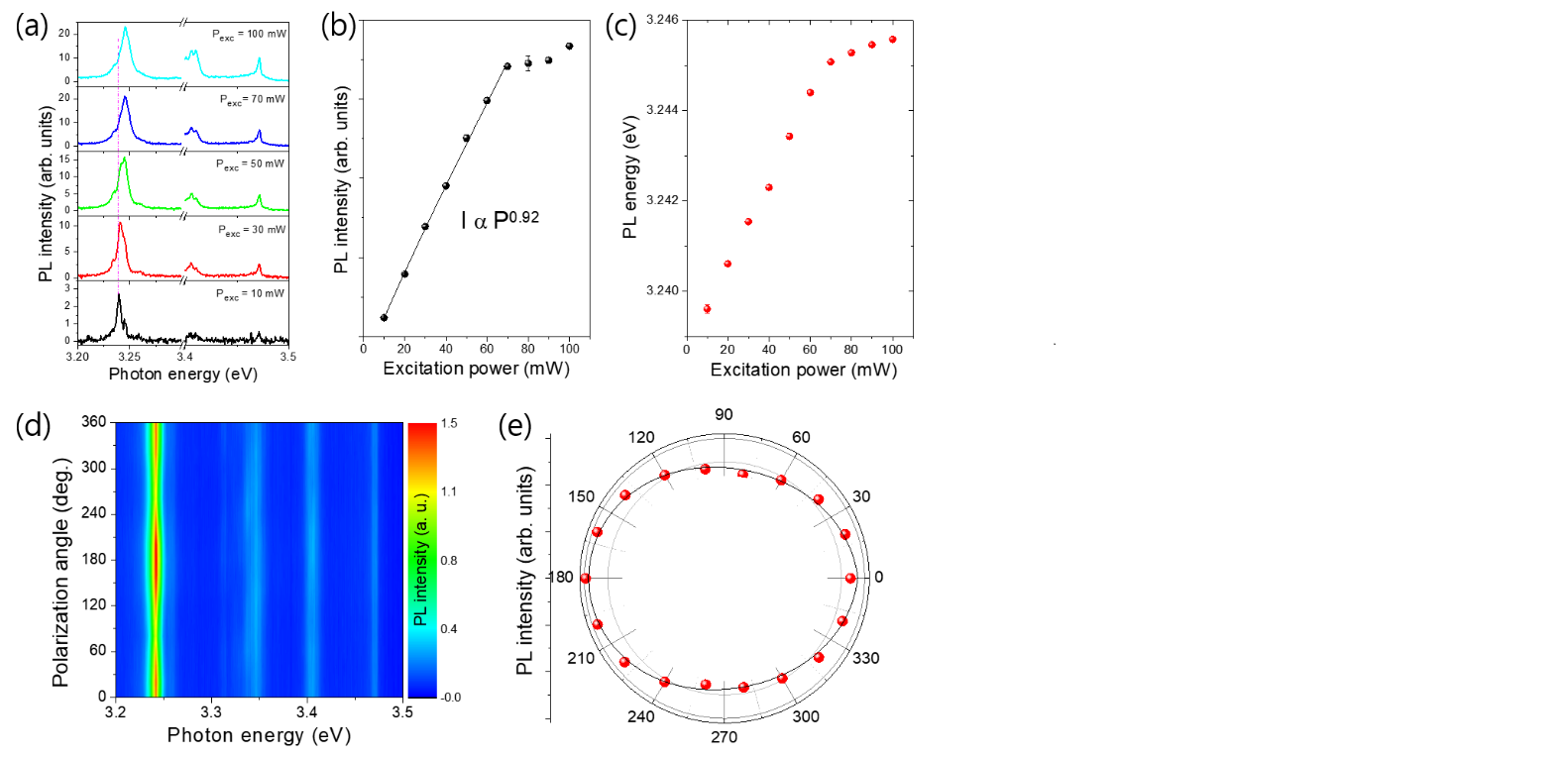


Figure S5. (a) Excitation power dependent PL spectra of the InGaN/GaN nanorods. The dotted magenta line is guide for eye indicating the PL energy shift with excitation power. (b) PL intensity variation as a function of excitation power. (c) PL peak energy as a function of excitation power. (d) PL spectra mapping of the InGaN/GaN nanostructures with different excitation angle. (e) PL intensity for the 3.24 eV emission as a function of polarization angle.
